# Targeting the pericyte antigen DLK1 with an alpha type-1 polarized dendritic cell vaccine results in tumor vascular modulation and protection against colon cancer progression

**DOI:** 10.3389/fimmu.2023.1241949

**Published:** 2023-10-02

**Authors:** Amanda L. McCormick, Trevor S. Anderson, Elizabeth A. Daugherity, Izuchukwu F. Okpalanwaka, Savanna L. Smith, Duke Appiah, Devin B. Lowe

**Affiliations:** ^1^ Department of Immunotherapeutics and Biotechnology, Jerry H. Hodge School of Pharmacy, Texas Tech University Health Sciences Center, Abilene, TX, United States; ^2^ Department of Public Health, School of Population and Public Health, Texas Tech University Health Sciences Center, Lubbock, TX, United States

**Keywords:** dendritic cells, colon cancer, anti-angiogenic therapy, DLK1, pericyte

## Abstract

Despite the availability of various treatment options, colorectal cancer (CRC) remains a significant contributor to cancer-related mortality. Current standard-of-care interventions, including surgery, chemotherapy, and targeted agents like immune checkpoint blockade and anti-angiogenic therapies, have improved short-term patient outcomes depending on disease stage, but survival rates with metastasis remain low. A promising strategy to enhance the clinical experience with CRC involves the use of dendritic cell (DC) vaccines that incite immunity against tumor-derived blood vessels, which are necessary for CRC growth and progression. In this report, we target tumor-derived pericytes expressing DLK1 with a clinically-relevant alpha type-1 polarized DC vaccine (αDC1) in a syngeneic mouse model of colorectal cancer. Our pre-clinical data demonstrate the αDC1 vaccine’s ability to induce anti-tumor effects by facilitating cytotoxic T lymphocyte activity and ablating the tumor vasculature. This work, overall, provides a foundation to further interrogate immune-mediated mechanisms of protection in order to help devise efficacious αDC1-based strategies for patients with CRC.

## Introduction

Metastatic colorectal cancer (CRC) remains a leading cause of cancer-related death in the United States despite treatment options that include surgery, chemotherapy, and targeted agents (1). CRC patients are typically stratified based on microsatellite instability (MSI) or microsatellite stability (MSS) phenotypes (2). MSI tumors yield tumor-specific neoepitopes (due to high mutational burdens) that facilitate anti-tumor T cell responses, particularly in combination with immune checkpoint blockade (ICB) (e.g., pembrolizumab, nivolumab) (3). However, such clinical responses are short-lived in up to a majority of MSI CRC patients (4). MSS tumors are not immunogenic by comparison, and ICB does not usually provide benefit to these individuals (4). Regardless, improved interventions like immunotherapies are desperately needed for all mCRC subtypes. A unifying theme across CRC subtypes is a reliance on tumor-derived blood vessels to mitigate scarcity of nutrients and oxygen as well as remove metabolic waste. Therefore, in addition to patient treatment inclusivity, a conceptual advantage to anti-angiogenic strategies involves specifically interfering with shared and required vascular targets that may also help minimize the onset of therapy resistance (5). Indeed, first- and second-line anti-vascular therapies (like the anti-VEGF antibody bevacizumab) have been established by the FDA for some time to provide mCRC patients improved survival (6). We have also demonstrated the safe and effective method of therapeutically inciting CD8+ T cells against vascular targets like endothelial cells and pericytes using dendritic cells (DCs) in an HLA-A2+ pre-clinical model of CRC (7).

Tumor-infiltrating DCs are crucial immune cells to target and destroy tumors in general, and indeed decades of work has resulted in the refinement of protocols to achieve various DC vaccines for cancer patients such as the FDA-approved drug sipuleucel-T for prostate cancer (reviewed in detail elsewhere (8)). These past development efforts (including CRC among other types) have led to notable improvements for select patients, but, on the whole, DC vaccine therapies have typically generated low objective response rates (9, 10). Although it is difficult to pinpoint prevailing issues across studies, the source/differentiation of DCs as well as antigen targets may play decisive factors in patient prognosis. Of the major circulating DC subsets in humans (i.e., conventional type 1 [cDC1], conventional type 2, and plasmacytoid DC [pDC]), cDC1s appear to be superior for eliciting anti-tumor immunity (11). Classically, cDC1s cross-present antigen to CD8+ T cells and attract/sustain cytotoxic T cells in the tumor microenvironment (TME) through the production of soluble mediators like CXCL9, CXCL10, and IL-12 (12, 13). Higher cDC1 infiltration into the TME also correlates with responsiveness to ICB and overall patient survival (13–15). Unfortunately, cDC1s are a rare human DC subset in circulation, curtailing their wider adoption into vaccines and forcing many DC vaccines to instead be derived/differentiated from bulk autologous monocytes. A potential improvement in this space involves differentiating monocytes to alpha-type I DCs (designated αDC1s). Typically, the process involves deriving mononuclear cells from the peripheral blood of humans (or bone marrow from mice) and culturing cells with an α-type-1-polarizing cocktail (containing TNF-alpha, IFN-alpha, IFN-gamma, IL-1-beta, and poly[I:C]). The resulting αDC1s fulfill 3 essential functions in the TME: a mature/antigen presenting cell phenotype, migratory abilities in response to secondary lymphoid organ chemokines, and elevated (rather than exhausted) IL-12 production. Notably, αDC1s heighten long-lived and antigen-specific cytotoxic T cells (16), and early phase clinical work supports their safety and therapeutic promise in cancer patients (17, 18). Yet, αDC1s have received less focus in syngeneic mouse modeling of cancer.

The nature of the current report is to more fully examine the strategy of a clinically-adopted αDC1 vaccine platform in a syngeneic mouse model against endogenous vascular components of the colon cancer microenvironment. Such mechanistic insights are anticipated to be translationally important since they could help direct refinements in αDC1 therapies and anti-angiogenic strategies in cancer patients. Here, we demonstrate the pre-clinical ability of an αDC1 vaccine to target the tumor-derived pericyte marker delta-like 1 homologue (DLK1) to achieve improved resolution of disease. Pericytes play a critical role in stabilizing endothelial tubes and promoting sprouting angiogenesis both at normal vascularized sites and within tumors (19). DLK1, in particular, is a transmembrane receptor that belongs to the epidermal growth factor-like family of proteins that regulates NOTCH signaling, which is important under settings of angiogenesis. Although DLK1 expression is widely apparent during embryonic development, expression in adult tissues is low (20, 21). Its overexpressed status in the TME likely helps preferentially localize immune-based activity that yields tumor-derived blood vessel destruction with acceptable/manageable safety (7, 17, 22, 23). Given such properties, DLK1 represents a promising anti-vascular target for immunotherapeutic strategies.

## Materials and methods

### Mice

Female 6-week-old C57BL/6J (strain # 000664) and OT-1 transgenic (strain # 003831) mice were purchased from The Jackson Laboratory and maintained in a pathogen-free animal facility in micro-isolator cages. All animals were handled following Institutional Animal Care and Use Committee (IACUC)-approved protocols in accordance with recommendations for the proper care and use of laboratory animals.

### Cell lines and culture

The murine colon adenocarcinoma cells MC38 (Kerafast) and Lewis lung carcinoma line (designated LLC) were maintained in complete media containing RPMI 1640 (Cytiva) supplemented with 10% heat-inactivated FBS (Corning), Penicillin/Streptomycin/Amphotericin B, 4 mM L-glutamine, 1 mM sodium pyruvate, and MEM non-essential amino acids (all from Thermo Fisher) at 5% CO_2_ in a 37°C humified incubator. Tumor cell lines were deficient in DLK1 expression (**Supplementary Figure 1A**) and confirmed to be mycoplasma-free prior to experiments.

### Peptides

The following H-2 Db/Kb-binding peptides were synthesized to > 95% purity (Genscript), dissolved in DMSO, and stored at -20°C until use: DLK1_162-169_ (FSGNFCEI), EphA2_682-689_ (VVSKYKPM) (24), and OVA_257-264_ (SIINFEKL).

### DC vaccine generation and analysis

Bone-marrow-derived cells were harvested from femurs/tibias of mice as previously described (25). Briefly, bulk cells were cultured in complete media with murine IL-4 (5 ng/mL) (Peprotech) and GM-CSF (10 ng/mL) (Peprotech) over a period of 5 days at 37°C with 5% CO_2_. All cells were then collected, washed, and subjected to magnetic bead positive selection in order to isolate CD11c+ DCs according to the manufacturer’s guidelines (cat# 130-125-835, Miltenyi Biotec). DCs were subsequently cultured in fresh complete media for 24 hr containing the following α-type-1-polarizing murine agents (all from Miltenyi Biotec unless otherwise stated) (16): 5 ng/mL IL-4 (Peprotech), 10 ng/mL GM-CSF (Peprotech), 10 ng/mL IFN-alpha, 1x10^3^ IU/mL IFN-gamma, 5 ng/mL TNF-alpha, 25 ng/mL IL-1-beta, and 10 μg/mL poly(I:C) (Sigma). The following day, non-adherent cells were collected, pelleted, and resuspended in Iscove’s Modified Dulbecco’s Medium (Thermo Fisher) at 1x10^6^ cells/mL. Peptides were added to cells at a final concentration of 10 μg/mL and cultured at 37°C with 5% CO_2_ for 3 hr. αDC1s were then pelleted, washed, and resuspended in PBS (1x10^6^ cells/100 μL) for individual animal injections.

In separate experiments, αDC1s were examined for the capacity to stimulate T cells *in vitro*. Splenocytes were first collected from OT-1 mice and CD8+ T cells positively selected with magnetic microbeads (Miltenyi Biotec). CD8+ T cells were then activated and expanded over a 4-day period as detailed elsewhere (26). More specifically, OT-1 CD8+ T cells were exposed to plate-bound anti-CD3 (clone 145-2C11, cat# BE0001-1) and anti-CD28 (clone 37.51, cat# BE0015-1) antibodies (Bio X Cell) for up to 48 hr in the presence of IL-2 and IL-7 (Peprotech) in complete media (enriched with beta-mercaptoethanol [BME] and insulin-transferrin-selenium [ITS] [Thermo Fisher]). Cells were collected, washed, and re-plated in fresh complete media containing IL-2, IL-7, BME, and ITS each day for up to 48 hr. Activated OT-1 CD8+ T cells were then co-cultured with SIINFEKL-pulsed αDC1s at a 2:1 E:T ratio in complete media in a 96-well plate at 37°C with 5% CO_2_. Positive control wells were stimulated with ConA (Sigma) at 2 μg/mL. After 48 hr, cell-free supernatants were collected and relative levels of IL-2 (cat# 555148, BD Biosciences) and IFN-gamma (cat# 551866, BD Biosciences) determined by ELISA. IL-12p70 secretion was determined in cell-free supernatant through ELISA (cat# 555256, BD Biosciences) after culturing αDC1s with plate-bound recombinant CD40L (cat# 50327-M07H, Sino Biological) for 48 hr.

αDC1s were also immunophenotyped by flow cytometry. Fc-gamma receptors were initially blocked using an anti-mouse CD16/32 antibody (clone 93, cat# 101301) (Biolegend) followed by specific surface staining with the following fluorochrome-conjugated anti-mouse monoclonal antibodies at 0.2 µg/mL (all from Biolegend): APC CD40 (clone 3/23, cat# 124611), APC CD80 (clone 16-10A1, cat# 104713), APC CD86 (clone GL-1, cat# 105011), APC CD25 (clone 3C7, cat# 101909), APC 1A-1E (clone M5/114.15.2, cat# 107613), APC H2-Db (clone KH95, cat# 111513), PE CD11c (clone HL3, cat# 553802) (BD Biosciences), APC CCR7 (clone 4B12, cat# 120107), APC CD83 (clone Michel-19, cat# 121509). A Zombie Aqua dye (cat# 423101, Biolegend) was also used to distinguish live cells from dead cells. Data was acquired on a BD LSR Fortessa (BD Biosciences) and analyzed using FlowJo software (version 10.6.2). Representative gating strategies are provided in **Supplementary Figure 2**.

### Tumor challenge model

Mice were randomized, assigned to treatment groups (containing 5 mice/cohort), and subcutaneously (s.c.) challenged with 5 x 10^5^ tumor cells (day 0). ICB was also incorporated with intraperitoneal (i.p.) injections of 200 μg of a PD-1 blocking antibody (clone RMP1-14, cat# BE0146) (Bio X Cell) on days 7, 10, and 13 post tumor challenge. In separate experiments, CD8+ T cells were depleted *in vivo* following i.p. injections of 200 μg of a CD8 blocking antibody (clone 2.43, cat# BE0061) (Bio X Cell) on days 3, 7, 10, and 13 after tumor inoculation. Tumor sizes and animal weights were obtained every 2-3 days. Tumor volumes were determined using digital Vernier calipers and the formula (a x b^2^) ÷ 2, where ‘a’ is the largest tumor diameter and ‘b’ represents the smallest tumor diameter measurement. At the end of the experiment, tumors and organs were excised and processed for further analysis.

### *Ex vivo* CD8+ T cell analysis

Splenocytes were collected from vaccinated or vaccinated/tumor-treated mice and CD8+ T cells purified through magnetic bead positive selection (cat# 130-117-044, Miltenyi Biotec). CD8+ T cells were then cultured for 24 hr in the presence of plate-bound anti-CD28 antibody, washed, and exposed to tumor cells or DLK1 162 peptide-pulsed αDC1s at a 20:1 E:T ratio in complete media at 37°C with 5% CO_2_. Positive control wells were stimulated with 2 µg/mL ConA. After 48 hr, cell-free supernatants were collected and relative levels of IFN-gamma determined by ELISA. Raw O.D. values were standardized to their respective ConA-stimulated treatments and data expressed as a function of normalized absorbance at 450 nm.

### Immunohistochemistry

Tissues were fixed in 10% neutral buffered formalin (Thermo Scientific) for 24 hr and dehydrated, cleared, processed into paraffin with a Leica automated tissue processor, and paraffin-embedded. FFPE tumor sections were cut at a thickness of 5 µM and mounted on positively-charged slides (Fisher Scientific). Tumor sections were washed sequentially with xylene, ethanol, PBS, and water and then incubated in boiling 10 mM sodium citrate for antigen retrieval (Fisher Chemical) for 15 min. Cooled sections were exposed to 3% H_2_O_2_ (Fisher Chemical) for 5 min, washed in PBS, and blocked for 30 min with 2.5% heat-inactivated horse serum (Thermo Fisher). Primary antibody incubation was carried out overnight at 4°C in a humified chamber with 1:100 anti-CD3e (clone SP7, cat# MA1-90582) (Thermo Fisher) or 1:50 anti-CD31 (clone SP38, cat# MA5-16337) (Thermo Fisher) antibodies. After washes with 0.5% Tween 20/PBS (PBST), an HRP-conjugated Horse-anti-Rabbit IgG secondary antibody (cat# MP-7401, Vector Laboratories) was applied to sections for 30 min at room temperature in the dark. Slides were again washed in PBST and stained/developed with a Metal Enhanced DAB Substrate kit (Thermo Scientific). Sections were counterstained with a 1:3 dilution of Hematoxylin 2 (Thermo Scientific) for 20 sec followed by successive washes in water, ethanol, and xylene before mounting in Acrymount (StatLab). Immunohistochemistry staining was imaged/quantified using Aperio ImageScope software (v.12.3.3.5048).

### Immunofluorescence

Harvested tumors or draining lymph nodes were embedded in Scigen Tissue-Plus O.C.T. compound (Thermo Fisher) and frozen over dry ice. Ten-micron tissue sections were adhered to positively-charged slides, fixed in 4% paraformaldehyde (Electron Microscopy Sciences), permeabilized with 0.2% Triton X-100 + 0.2mM glycine (Fisher Scientific), and blocked with 5% FBS in 0.2% Triton X-100. Sections were then exposed to primary antibodies against CD31 (1:500) (clone MEC 13.3, cat# 550274) (BD Biosciences), CD8 (1:200) (clone 208, cat# MA5-29682) (Thermo Fisher), PDGFR-β (1:100) (clone G.290.3, cat# MA5-15143) (Thermo Fisher), or CD11c (1:100) (clone EPR21826, cat# ab219799) (Abcam) followed by incubations with donkey anti-rat 488 (cat# 712-545-153) or donkey anti-rabbit 594 (cat# 711-585-152) secondary antibodies (both from Jackson ImmunoResearch). For staining apoptotic cells, 5-micron sections from paraffin-embedded tumors were prepared as described above. After sodium citrate antigen retrieval, tissue sections were incubated with TUNEL reagents according to the manufacturer’s protocol (cat# 25870, Cell Signaling Technology). Coverslips were then applied over an anti-fade mounting medium containing DAPI (Vector Laboratories) and processed for immunofluorescence microscopy using a MICA Microhub microscope (Leica). Quantification of specific staining was later performed using the Leica Application Suite X software (v.1.4.4.26810).

### Quantitative PCR

Select cell lines, tumors, and organs were processed in TRIzol (Thermo Fisher) and total RNA isolated using the DirectZol RNA Miniprep Kit (Zymo Research). cDNA was next generated with the SuperScript IV First-Strand Synthesis System (Thermo Fisher). Various gene targets were amplified (using validated qPCR primers [Origene] [**Supplementary Table 1**]) and quantitated using a Fast SYBR Green Master Mix (Applied Biosystems) on a StepOnePlus real-time PCR thermocycler (Applied Biosystems) – with specific amplicons being corroborated through melt curve analysis. All qPCR reactions were performed in duplicate in a 96-well PCR plate (Bio-Rad) using the following cycling conditions: initial denaturation at 95°C for 20 min followed by 35 cycles at 95°C for 3 min and 60°C for 30 min. Gene expression levels were finally normalized to GAPDH and relative fold changes determined using the 2^-ΔΔCt^ method (27).

### Statistical analysis

Select results were analyzed with either unpaired t-test or one-way ANOVA + *post-hoc* pairwise comparisons using GraphPad Prism (version 9.3.1). Experiments analyzing tumor burden *in vivo* were performed using repeated measures ANOVA across all time points. An autoregressive with heterogenous variance covariance matrix was selected based on Bayesian information criterion for the model. To control the false positive rate in *post-hoc* analyses, *P* values were adjusted for multiple comparisons with Dunnett’s test using SAS 9.4 4 (SAS Institute, Inc.). Mean differences with a *P* value < 0.05 were considered statistically significant. Data presented are representative of at least 3 independent experiments.

## Results

### Differentiation and characterization of murine alpha type I DCs

**Figure 1:**
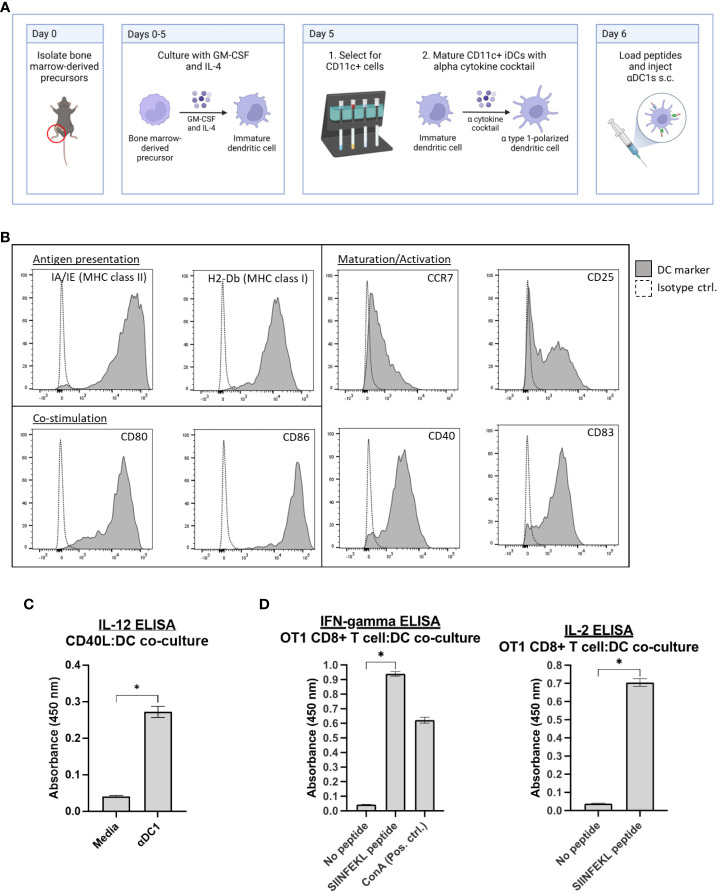
Characterization of αDC1s. **(A)** Typical schema for DC generation and αDC1 polarization. **(B)** αDC1s were analyzed for markers of antigen presentation, DC maturation/activation, and co-stimulation by flow cytometry. **(C)** IL-12p70 secretion was determined by ELISA after αDC1 incubation with recombinant CD40L. **(D)** αDC1s loaded with or without SIINFEKL peptide and co-cultured with activated OT1 CD8+ T cells for 48 hours. Cell-free supernatant was analyzed for IFN-gamma and IL-2 secretion by ELISA. *P < 0.05, bars ± STDEV.

Bone-marrow-derived cells were isolated from C57BL/6 mice and, over several days in culture, CD11c+ DCs differentiated toward an αDC1 phenotype and loaded with relevant peptides (outlined in **Figure 1A**). To initially assess the potential for these cells to mediate T cell interactions and activation in secondary lymphoid tissues, αDC1s were examined by flow cytometry and confirmed to exhibit properties for antigen presentation (MHC Class I and II molecules), maturation (CD25, CCR7, CD83, and CD40), and co-stimulation (CD80 and CD86) (**Figure 1B**). IL-12p70 secretion capability was also demonstrated by ELISA after exposing αDC1 cells *in vitro* to recombinant CD40L, which is an important co-stimulatory molecule expressed by activated lymphocytes such as T cells that helps stimulate anti-tumor immune responses (**Figure 1C**) (28). We next determined αDC1 functionality by co-culturing SIINFEKL-pulsed αDC1s with activated OT-1 CD8+ T cells *in vitro* over 48 hrs, and cell-free supernatant was assessed by ELISA. Ultimately, **Figure 1D** demonstrates αDC1’s role in promoting CD8+ T cell secretion of IFN-gamma and IL-2 (cytokines instrumental to T cell cytotoxicity and persistence, respectively (29, 30) in a peptide-specific manner. In all, these data help confirm αDC1s suitability as antigen presenting cells (APCs).

### αDC1-DLK1 vaccination safely promotes improved colon cancer protection

**Figure 2:**
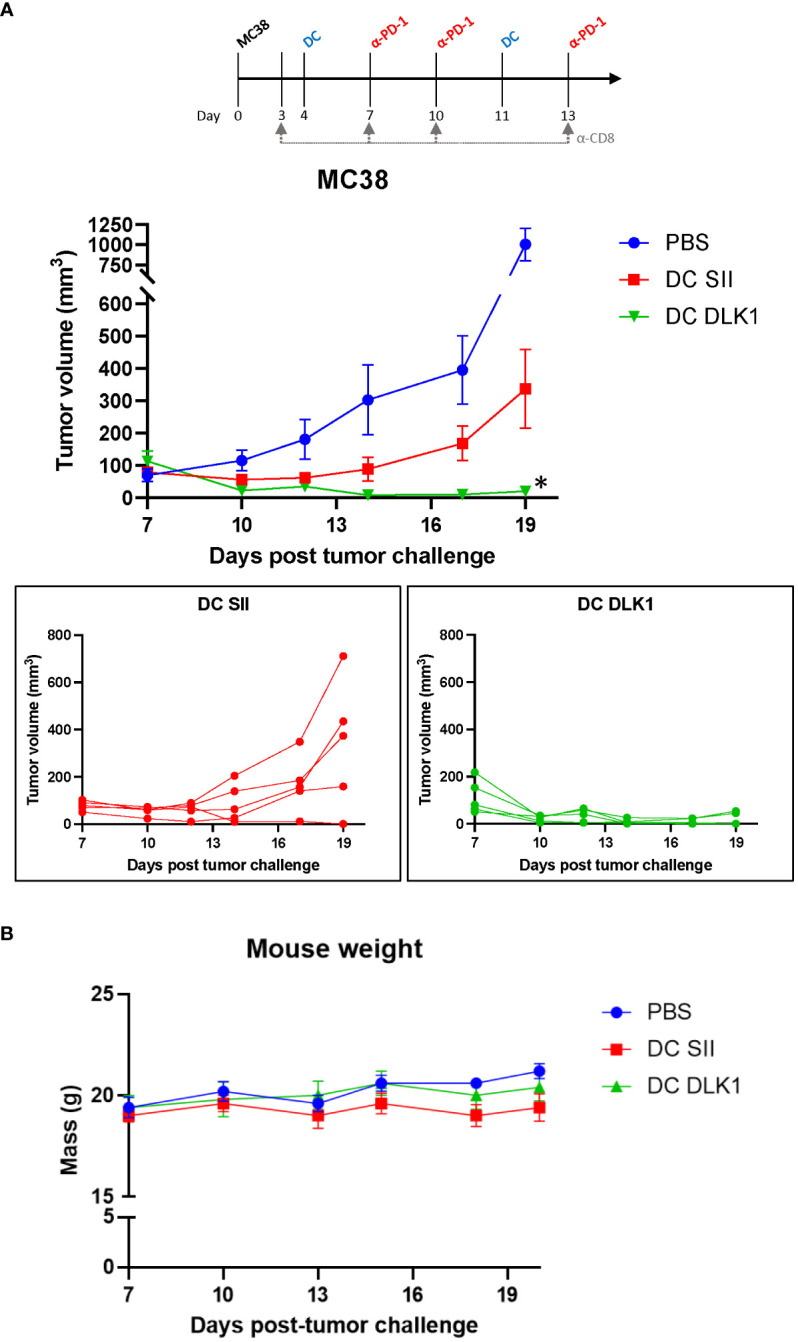
Therapeutic vaccination with anti-DLK1 αDC1 alongside anti-PD1 blockade enhances protection against MC38 tumor challenge. **(A)** Tumor growth kinetics of MC38-bearing mice treated with the αDC1-DLK1 vaccine on days 4 and 11, with anti-PD1 antibody infused on days 7, 10, and 13. **(B)** Animal weights were tracked for the duration of the experiment as an indicator of therapy safety. *P < 0.05 compared to all treatment cohorts, bars ± SEM.

DLK1 represents a suitable vascular target for vaccine purposes as it is upregulated on pericytes in many solid vascularized tumors and persists at low levels in vital tissues (**Supplementary Figure 1A**) (21). To help ensure specific targeting of tumor-derived blood vessels, we confirmed by PCR that the MC38 tumor cell line does not detectably express DLK1 but is present in MC38 *in vivo* tumors (**Supplementary Figure 1A**). We have also previously documented by IF that DLK1 is expressed by pericytes in the MC38 tumor microenvironment (TME) (7). Therefore, using NetMHC 4.0 (31, 32), we selected a peptide (FSGNFCEI) from the full-length sequence of murine DLK1 predicted to bind with strong affinity to MHC Class I (H-2 Db) on DCs. Mice were challenged with MC38 cells (day 0) and began receiving αDC1 vaccine treatments s.c. every 7 days once tumors were palpable on day 4 (**Figure 2A**). To help counter TME-instigated immunosuppression, animals also received i.p. injections of an anti-PD-1 antibody on days 7, 10, and 13. Vaccinated mice yielded DC accumulation (based on CD11c+ IF) in the tumor draining lymph node, helping suggest the location of T cell interaction/activation with antigen-charged αDC1 cells (a classical phenomenon in line with other studies incorporating DC vaccines – [ (33, 34) as examples] (**Supplementary Figure 3**). Notably, PD-1 blockade had no observable protective effects when administered as a monotherapy (**Supplementary Figure 4**). Overall longitudinal tumor sizing indicated a protective effect in mice receiving irrelevant SIINFEKL peptide-pulsed αDC1s (designated αDC1-SII) compared to PBS treatments, potentially a result of αDC1s ability to naturally process/present tumor debris and generate tumor cell-specific immunity (16, 35). However, under our modeling, DLK1 peptide-pulsed αDC1s mediated superior protection versus all treatment cohorts by directing immune-based responses against DLK1+ stromal components. Interestingly, in separate experiments, αDC1-DLK1 immunization performed as well as an αDC1 vaccine incorporating the VVSKYKPM peptide derived from EPHA2. Based on target expression profiles, αDC1-EphA2 likely mediated specific immune responses against both MC38 tumor cells (**Supplementary Figure 1B**) and tumor-derived endothelial cells to provide maximum modeling protection (7). These data are important since they help validate that αDC1-DLK1 therapy could, under certain scenarios, provide effective tumor control by initially inspiring immune responses to a conserved vascular target that is not expressed by tumor cells.

The general safety of αDC1-DLK1 vaccination was also assessed by analyzing mouse weights over time. **Figure 2B** demonstrates that our therapeutic regimen did not adversely impact animal health based on minimal weight deviations (i.e., not exceeding 20% reduction in body mass) from PBS control treated animals. Additionally, heart, lung, and kidneys were harvested from euthanized mice at the end of the study and examined by H&E, but no histological abnormalities like immune cell infiltration, tissue disturbance/destruction, or vascularity were observed (**Supplementary Figure 5**). Such results help support the safe and improved efficacy of an αDC1-inspired immune response initially directed toward vascular targets like DLK1 in the TME.

### Therapeutic immunization against DLK1 enhances intratumoral cytotoxic CD8+ T cells

**Figure 3:**
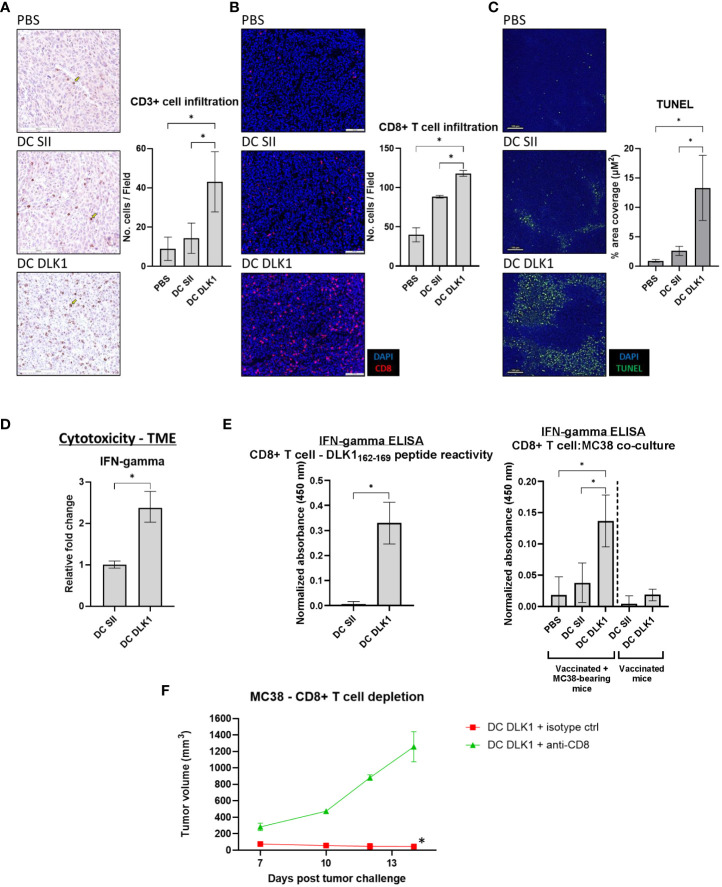
Induction of a Type-I prone intratumoral T cell responses following αDC1-DLK1 vaccination. **(A)** IHC analysis of CD3+ cells in MC38 tumors treated with an αDC1-DLK1 vaccine. 500 x 500-micron field views were randomly generated from tumors for each cancer lesion in an experimental group and total positive events averaged. **(B)** CD8+ T cell infiltration examined by IF. 650 x 550-micron fields were randomly selected from individual tumors in each experimental group and total positive events averaged. **(C)** *In situ* detection of apoptotic cells in the TME by TUNEL assay. **(D)** qPCR analysis of the CD8+ T cell cytotoxicity marker IFN-gamma in MC38 tumors. Gene expression was normalized to the housekeeping gene GAPDH and presented as relative fold-change. **(E)** CD8+ T cells were isolated from treated mice (αDC1 vaccine or αDC1 vaccine/MC38 tumor challenge) and co-cultured with either DLK1 peptide or MC38 tumor cells for 48 hr. IFN-gamma secretion was analyzed by ELISA and absorbance results normalized to ConA responses in control wells. **(F)** CD8+ T cell depletion study in mice receiving a neutralizing antibody on days 3, 7, 10, and 13. Tumor growth kinetics were calculated between treated cohorts. *P < 0.05, bars ± SEM. Yellow arrow inset distinguishing a stained cell of interest.

Tumors excised at day 19 were examined by IHC for total CD3+ cells in order to estimate total T cell abundance in cancer lesions. **Figure 3A** demonstrates that αDC1-DLK1 vaccination elicited a greater than 3-fold increase in CD3+ cells when compared to αDC1-SII animals (43 [αDC1-DLK1] *vs*. 14 [αDC1-SII] CD3+ cells per field). These results were corroborated more sensitively by IF in **Figure 3B**, indicating similar trends with significant increases of intratumoral CD8+ T cells in mice following αDC1-DLK1 treatment (118 [αDC1-DLK1] *vs*. 88 [αDC1-SII] CD8+ T cells per field). Additionally, these properties with αDC1-DLK1 vaccination correlated to greater detection of apoptotic cells in the TME when tumor lesions were stained *in situ* using a TUNEL assay (**Figure 3C**). Since we are performing transplantable tumor models, tumors are grown s.c. and do not invade the underlying healthy tissue. Therefore, tumors are easily excised and prepared so that the data presented are giving an account of what is occurring in the TME (i.e., there is little to no healthy tissue available to assess).

Whole tumor tissues were also processed/assessed by qPCR for general immune-driven markers. In comparison to αDC1-SII, αDC1-DLK1 treatment resulted in a significantly enhanced IFN-gamma expression profile (**Figure 3D**) with marked reductions in immunosuppressive factors like PD-L1, CCL2, and CSF1 (**Supplementary Figure 6**). Given the potential therapeutic role of CD8+ T cells in our model, CD8+ T cells were isolated from spleens of treated animals to determine antigen specificity. As the αDC1-DLK1 vaccination readily induced systemic CD8+ T cell responses against the DLK1_162-169_ peptide, reactivity against MC38 cells was only evident in animals also experiencing protection against MC38 tumor challenge *in vivo* (**Figure 3E**). Lastly, MC38 challenged mice were depleted of CD8+ T cells during the course of αDC1-DLK1 vaccination (**Figure 2A**). **Figure 3F** confirms that CD8+ T cells were critically required to mediate and prevent MC38 progression since animals rapidly succumbed to tumor challenge in the absence of a functioning cytotoxic T cell response. Altogether, the observed anti-tumor effects observed in αDC1-DLK1 vaccinated mice (**Figure 2A**) are presumably aided by improved infiltrating cytotoxic CD8+ T cells (cross-primed against various TME-associated antigens) functioning in a Type-I shifted immunological environment.

### Colon cancer-derived blood vessels are impaired post αDC1-DLK1 vaccination

**Figure 4:**
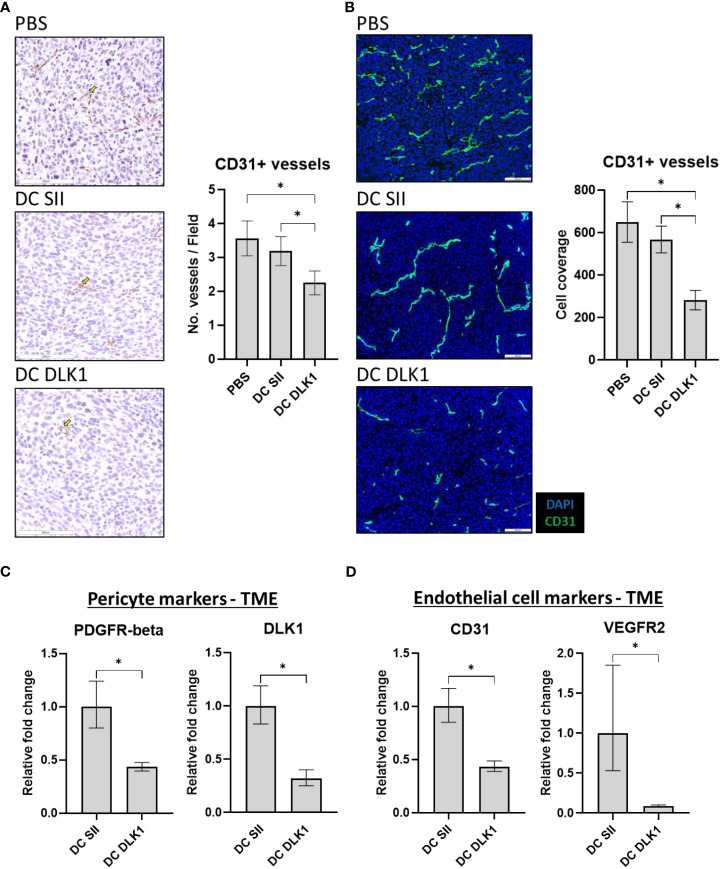
αDC1-DLK1 therapeutic immunization alters MC38-derived vascular density and total content. **(A)** IHC analysis of CD31+ vessels following treatment. 500 x 500-micron field views were generated randomly from each tumor lesion of a group and total stained cells averaged. **(B)** IF staining and analysis of CD31+ vessel coverage in MC38 tumors. 650 x 550-micron fields were randomly selected from individual cancer lesions in a treatment group and total positive events averaged. Relative mRNA expression of markers associated with pericytes **(C)** and endothelial cells **(D)** in MC38 tumors by qPCR. Gene expression was normalized to the housekeeping gene GAPDH and presented as relative fold-change. *P < 0.05, bars ± SEM. Yellow arrow inset demarking a stained cell of interest.

Post-treatment tumors were examined for vascular property disturbances to verify our vaccine strategy of initiating anti-vascular immune responses. αDC1-DLK1 vaccination potentiated significant reductions in CD31+ vessel density when determined by IHC (**Figure 4A**) and subsequently confirmed by IF (**Figure 4B**). Such imaging analysis was further supported by quantifying expression of blood vessel related targets through qPCR. As outlined in **Figures 4C, D**, αDC1-DLK1 treatment led to substantial losses in markers associated with pericytes (PDGFR-beta and DLK1) and endothelial cells (CD31 and VEGFR2). In tandem with biased Type-I immunity, immune-based reactions against vascular target such as DLK1 likely help account for tumor regression and protection in our described colon cancer model.

Given the need to adhere to MHC haplotypes (since the DLK1 peptide is predicted to bind H-2 Db) and limited CRC transplantable models in C57BL/6 mice, we confirmed the anti-vascular effects of our αDC1-DLK1 vaccination scheme using a separate *in vivo* model incorporating LLC cells. The LLC cell line is characterized as poorly immunogenic based on factors such as down-regulated MHC class I (36) and resistance to ICB (36, 37), which somewhat recapitulate characteristics of CRC (38, 39). Mice were s.c. challenged with LLC cells and therapeutically vaccinated and provided ICB treatment as detailed in **Figure 2A**. Similar to effects observed with MC38, αDC1-DLK1 vaccination led to significant size reductions in LLC tumor lesions (**Supplementary Figure 7A**) that were also deficient in CD31+ vessels by IHC and qPCR when compared to control treated cohorts (**Supplementary Figure 7B**).

Overall, our results help establish the utility of safely and effectively instituting cytotoxic T cells against tumor-derived blood vessels using αDC1s in the colon cancer microenvironment. This pre-clinical syngeneic model should now provide an opportunity to more fully examine immune-mediated reactions against stromal content within the tumor microenvironment to better understand settings of tumor relapse and progression.

## Discussion

Like other tissues in the body, vascularized cancers rely on the formation and maintenance of blood vessels in the TME (40, 41). Selectively targeting crucial angiogenic pathways (by way of small molecule or immunotherapeutic interventions) can disrupt tumor cell survival and dissemination and prolong CRC patient survival (42). An additional advantage to the approach involves broad applicability to mediate disease regression across solid tumor types, irrespective of cancer cell genetic heterogeneity, given the required and conserved nature of many vascular targets like VEGF/VEGFRs (43). However, a sensitized TME usually develops resistance to anti-angiogenic therapies that limits long-term efficacy (44). There is, therefore, a crucial need to explore and understand additional interventions (including new blood vessel targets and combinatorial regimens) that can safely impart durable vascular destabilizing strategies.

The promising clinical outcomes observed with ICB in MSI CRC help substantiate the approach of provoking immune effector cell activity within the TME (45). DCs, in particular, are necessary mediators for inciting Type-I-prone CD8+ T cells, and DC vaccines represent a valid immunotherapeutic strategy to direct/amplify antigen-specific immunity against tumor blood vessels (8). Although various isolation and *ex vivo* processes exist to provide patients an autologous cell preparation, underwhelming responses in cancer patients to date could involve an inability to infuse DCs with robust APC activity, highlighting the need for preclinical development and clinical investigation of alternative DC vaccine platforms (46, 47). As one such approach, αDC1s are capable of stimulating robust anti-tumor immune responses by facilitating the cross-presentation of tumor antigens, leading to the generation of superior tumor-specific T cell responses *in vitro* across cancer types that include melanoma (16), prostate cancer (48), and chronic lymphocytic leukemia (49). Early-phase clinical trials have likewise shown promising results in melanoma (17, 50), CRC (18), and malignant glioma (51) with clinical investigations underway in triple negative breast cancer (NCT05539365), ovarian cancer (NCT03735589), and renal cell carcinoma (NCT05127824). However, refinements to the approach may be potentially hindered since αDC1 assessments have received less attention in pre-clinical cancer models.

Pericytes play a critical role in stabilizing blood vessels and promoting angiogenesis within tumors, and also harbor antigens against which anti-cancer vasculature therapeutics can be designed (19). By targeting the pericyte antigen DLK1, αDC1 vaccination led to significant changes in colon cancer vascular density, marked overall by reduced pericyte (PDGFR-beta and DLK1) and endothelial cell (CD31 and VEGFR2) markers. Interestingly, endothelial cells remaining in the TME following DC DLK1 vaccination were still associated with pericytes (**Supplementary Figure 8**), implying the selection of vessels with a mature phenotype (52). These observations suggest that αDC1 immunization could help facilitate a more organized blood vessel system (i.e., instituting “vessel normalization”) (at least temporarily) to enhance the perfusion and function of anti-tumor agents, although these findings would need to be confirmed and better understood in future studies (53–55). Indeed, DLK1 vaccination also promoted enhanced deposits of Type-1 skewed CD8+ T cells that likely contributed to durable anti-tumor responses through epitope spreading in a majority of mice (see **Figure 3E**). Inspiring these diverse immune-based effects against a vascularized primary lesion would be important under settings where the TME eventually develops resistance to anti-vascular effects or progressive disease adopts growth patterns through non-angiogenic phenomena like vessel co-option (56). The nature of this report is similar to a previous study that utilized IL-4/GM-CSF differentiated DCs (i.e., immature DCs) to enhance protection against MC38 challenge by prophylactically stimulating immunity against murine EphA2 (expressed by both MC38 and endothelial cells) (57). Interestingly, we were capable of corroborating protective effects using αDC1s loaded with an EphA2 peptide but under therapeutic settings – a more clinically relevant scenario (**Supplementary Figure 4B**). Our modeling scheme, though, is unique since we are not aware of other groups focusing on αDC1 anti-angiogenic vaccination (+ ICB) within the constraints of colon cancer.

Combining anti-angiogenic strategies with ICB may offer promising avenues for effective treatment strategies to overcome inherent/acquired resistance against either agent alone (52). Our current study mirrors (to some degree) the observation of limited ICB effectiveness in MSS patients (58) since anti-PD-1 monotherapy did not afford anti-tumor benefits (**Supplementary Figure 4**). Yet, directing αDC1 vaccines against endogenous DLK1 on tumor-derived stromal cells was beneficial to mice only in combination with anti-PD-1 blockade (**Figure 2A**; **Supplementary Figure 4**). Stromal cells like fibroblasts and endothelial cells within the TME can play a critical role in influencing ICB potency. For example, in a murine model of MSS CRC, FAP-expressing fibroblasts discouraged anti-tumor responses to anti-PD1 treatment by instilling an immunosuppressive environment through recruitment of MDSCS (59). Angiogenic growth factors may also drive T cell exhaustion within the TME, which negates the positive benefits of ICB. In one perspective, VEGF helped directly drive T cell exhaustion in MSS CRC patients through the expression of the transcription factor TOX (60). Concurrently disabling PD-1/VEGF interactions with their cognate ligands restored effector T cell function and killing of MSS CRC cells. Such findings are also supported by a separate study reporting benefits to combining regorafenib (an FDA approved multikinase inhibitor with anti-angiogenic and immunomodulatory effects) and anti-PD1 antibody. Mice receiving both agents controlled MSS CRC burden (even after therapy discontinuation), whereas, anti-PD1 or regorafenib monotherapies had negligible lasting effects (61).

Altogether, our work here complements recent clinical efforts establishing that αDC1 vaccines can provide improved resolution of disease by initiating immune effector cells against the tumor-derived vasculature (17). It remains possible that despite upregulated vascular antigens in the TME, αDC1-based strategies foster vascular-specific memory T cell responses that could (over time) incite unmanageable adverse events in patients. However, this concern has not manifested in clinical evaluations (17) and may be thwarted in part by mechanisms of resistance for vessels at non-tumor sites (62, 63). We are currently focused on unraveling immune-mediated mechanisms involved in vascular changes induced by αDC1-DLK1 vaccination, particularly under orthotopic settings of colon cancer progression since s.c. tumor modeling may overestimate anti-vascular effects (due to increased angiogenesis) or not accurately reflect the CRC TME of patients (38, 64). Identifying the key dynamics of protective immune responses pre-clinically will likely play an important role in helping inform future work with αDC1 vaccines in individuals with vascularized disease like CRC.

## Data availability statement

The datasets presented in this article are not readily available. Requests to access the datasets should be directed to Dr. Devin Lowe, devin.lowe@ttuhsc.edu.

## Ethics statement

The animal study was approved by Texas Tech University Health Sciences Center IACUC. The study was conducted in accordance with the local legislation and institutional requirements.

## Author contributions

Designing research studies: AM, DL. Conducting laboratory experiments: AM, TA, ED, IO, SS. Acquiring data: AM, TA, ED, IO, SS. Analyzing data: AM, DA, DL. Writing/editing the manuscript: All authors. All authors contributed to the article and approved the submitted version.

## Supplementary material

Supplementary Figure 1 Variable expression of DLK1 and EphA2 in MC38 tumor cells. **(A)** Qualitative PCR and qPCR demonstrating the absence of DLK1 expression in tumor cell lines but presence in the TME. **(B)** Qualitative PCR and IF confirming EphA2 expression in MC38 cells.

Supplementary Figure 2 Flow cytometry gating strategy for αDC1 immunophenotyping. CD11c+ αDC1 cells were prepared and stained for flow cytometry according to the Materials and Methods section. Up to 100,000 events were captured, a time gate applied to collected events (to minimize aberrant sample collection and/or electronic noise), and successive gating employed to obtain viable single cells. Data are presented as normalized frequency of a particular DC marker relative to an isotype control.

Supplementary Figure 3 αDC1 vaccinated mice maintain DCs in tumor draining lymph nodes. Mice were vaccinated with αDC1s, challenged with MC38 tumors, and draining lymph nodes excised and stained for DC accumulation by IF. Representative images indicate DC presence in tumor draining lymph nodes of αDC1 vaccinated mice.

Supplementary Figure 4 PD-1 blockade is necessary for therapeutic efficacy of αDC1-DLK1 vaccination in MC38. **(A)** No anti-tumor effects were observed when an αDC1-DLK1 vaccine was administered as a monotherapy. **(B)** Inclusion of anti-PD-1 antibody in the therapeutic regime (see ) yields reduced tumor burdens when animals received an αDC1 vaccine loaded with a DLK1 or EphA2 peptide. Bars ± SEM.

Supplementary Figure 5 αDC1-DLK1 vaccination does not impair the structural integrity of vital tissues. Heart, lung, and kidneys were harvested from animal cohorts and processed for H&E staining. Representative images help confirm that αDC-based treatments did not result in observable abnormalities of normal tissues.

Supplementary Figure 6 Markers associated with immunosuppression are downregulated in αDC1-DLK1 treated mice. Relative PD-L1, CCL2, and CSF1 mRNA levels were assessed by qPCR in the MC38 TME and demonstrated to be significantly altered following αDC1-DLK1 vaccination. Gene expression was normalized to the housekeeping gene GAPDH and presented as relative fold-change. **P* < 0.05, bars ± SEM.

Supplementary Figure 7 anti-DLK1 αDC1 vaccination affords protective and anti-vascular effects against LLC tumor challenge. **(A)** Longitudinal tumor sizes of LLC-bearing mice treated with the αDC1-DLK1 vaccine on days 4 and 11 and anti-PD1 antibody provided on days 7, 10, and 13. **(B)** At study end, tumors were excised and stained for the presence of CD31+ vessels by IHC and qPCR. **P* < 0.05 compared to all treatment cohorts, bars ± SEM. Yellow arrow inset demarking a stained cell of interest.

Supplementary Figure 8 Residual vessels are associated with pericytes following anti-DLK1 αDC1 therapy. In preliminary assessments, vessel phenotype was assessed by IF in tumors subsequent to αDC1-based treatments. anti-DLK1 αDC1 vaccination resulted in CD31+ endothelial tubes associated with PDGFR-beta+ pericytes.

Supplementary Table 1 qPCR Primer Pairs.
