# Supplementary material for: Targeting the pericyte antigen DLK1 with an alpha type-1 polarized dendritic cell vaccine results in tumor vascular modulation and protection against colon cancer progression
Source: Front Immunol. 2023 Oct 2;14:1241949. doi: 10.3389/fimmu.2023.1241949 (PMC10578441; doi:10.3389/fimmu.2023.1241949)
Supplement: Supplementary Figure 1 — Variable expression of DLK1 and EphA2 in MC38 tumor cells. (A) Qualitative PCR and qPCR demonstrating the absence of DLK1 expression in tumor cell lines but presence in the TME. (B) Qualitative PCR and IF confirming EphA2 expression in MC38 cells. [file Presentation_1.pdf]

Supplementary Figure 1

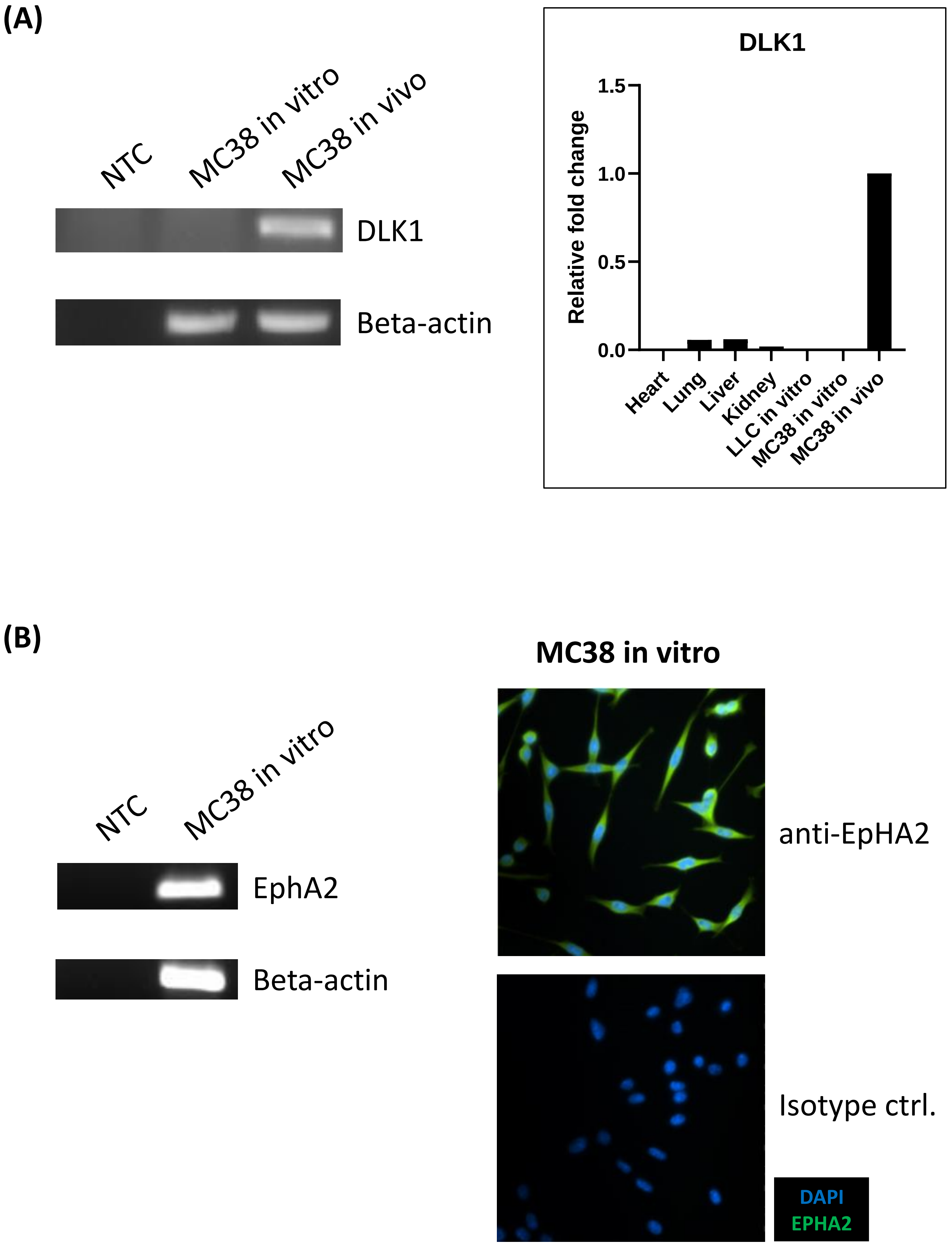

Supplementary Figure 2

Purified/matured αDC1s (ex vivo)

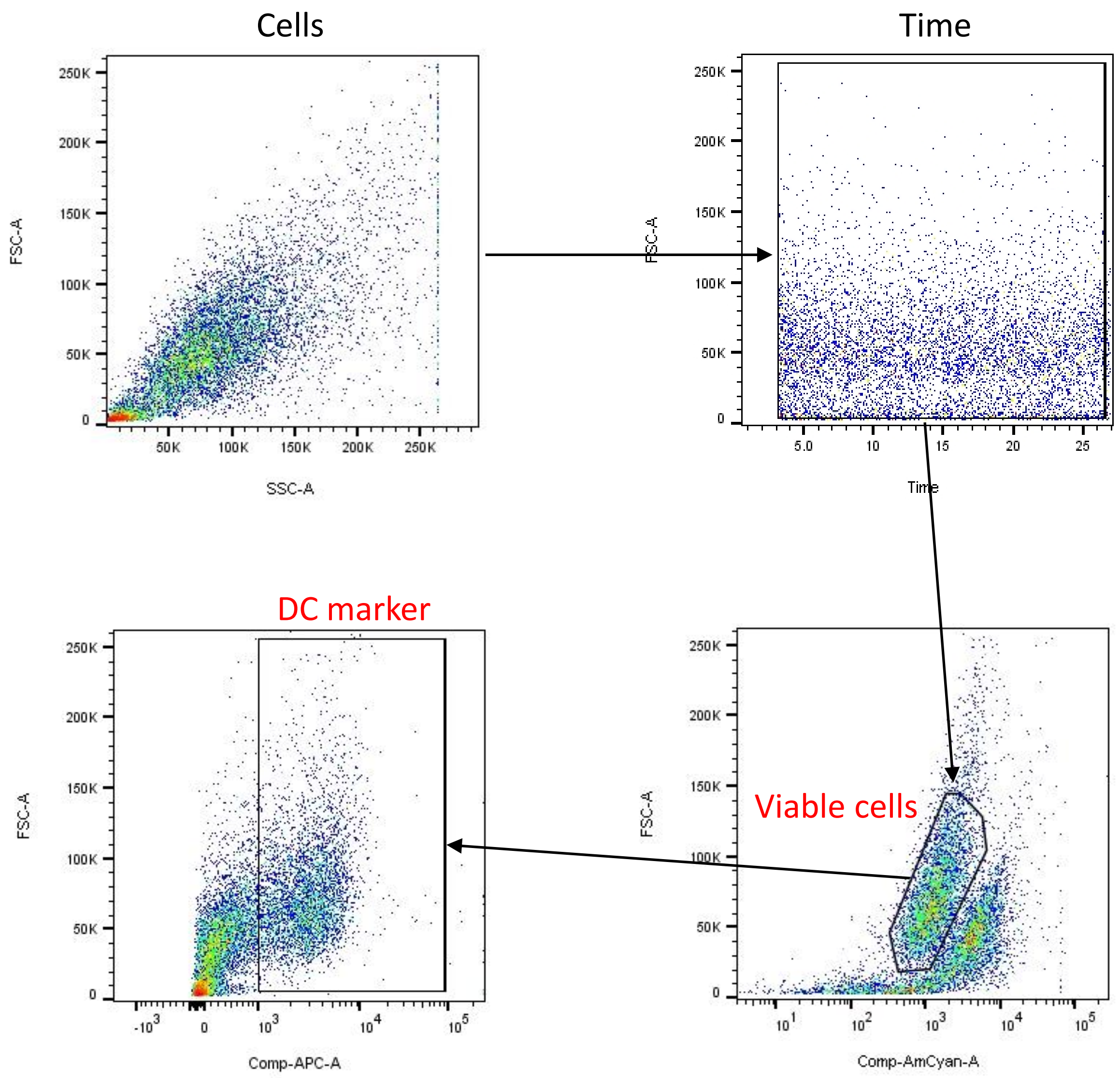

Supplementary Figure 3

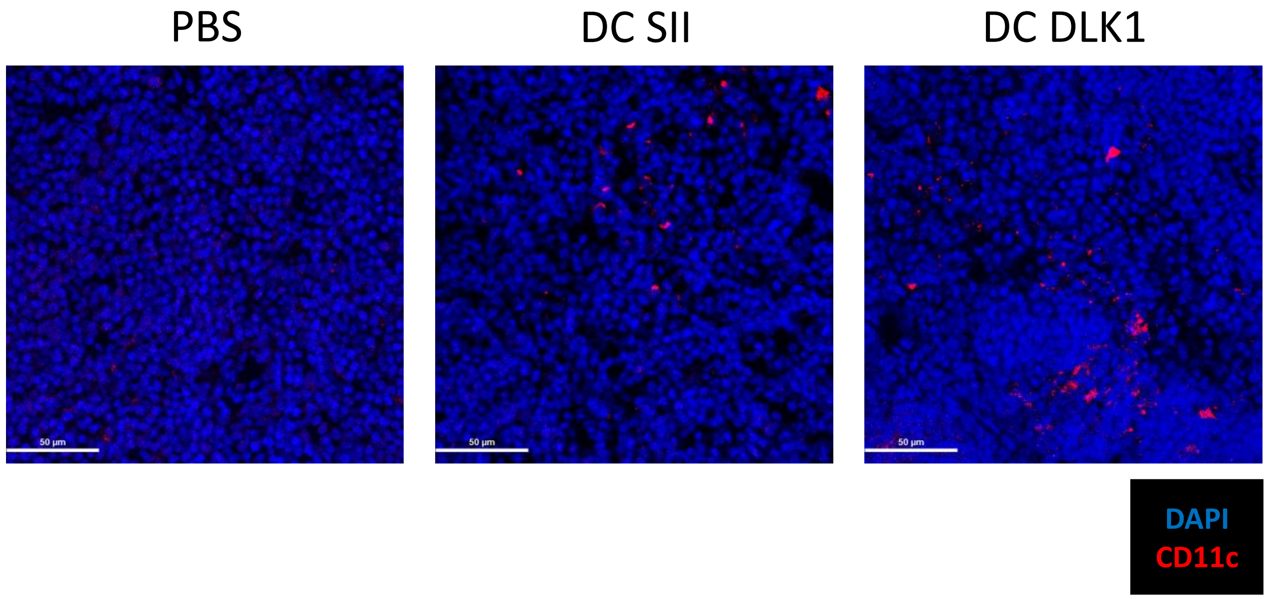

Supplementary Figure 4

(A)

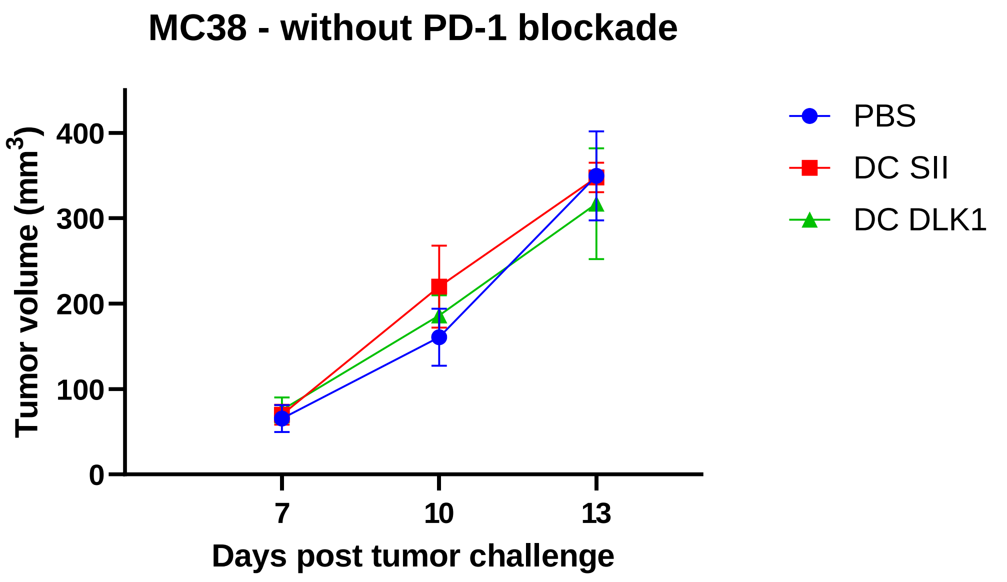

(B)

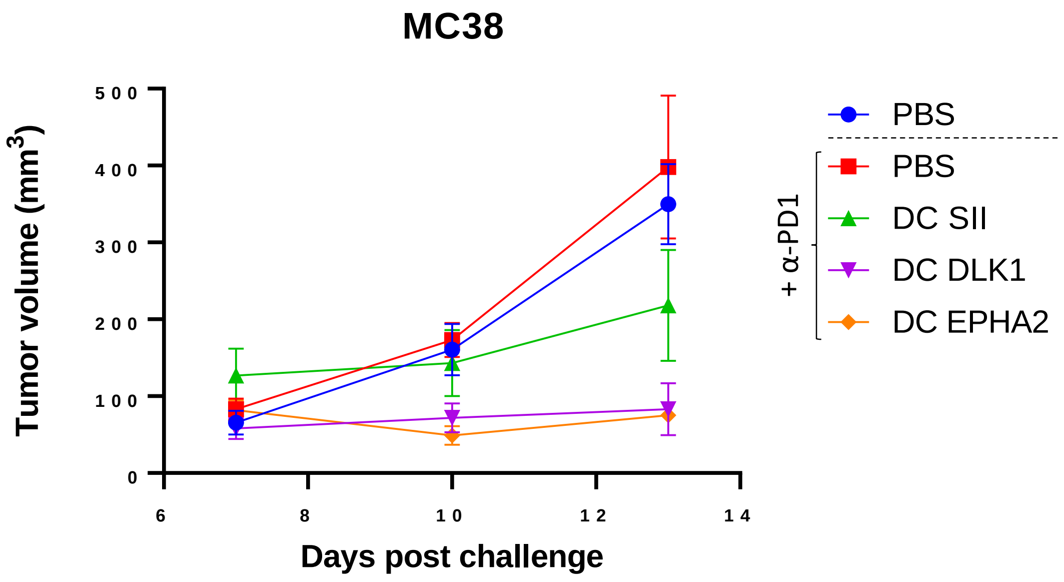

Supplementary Figure 5

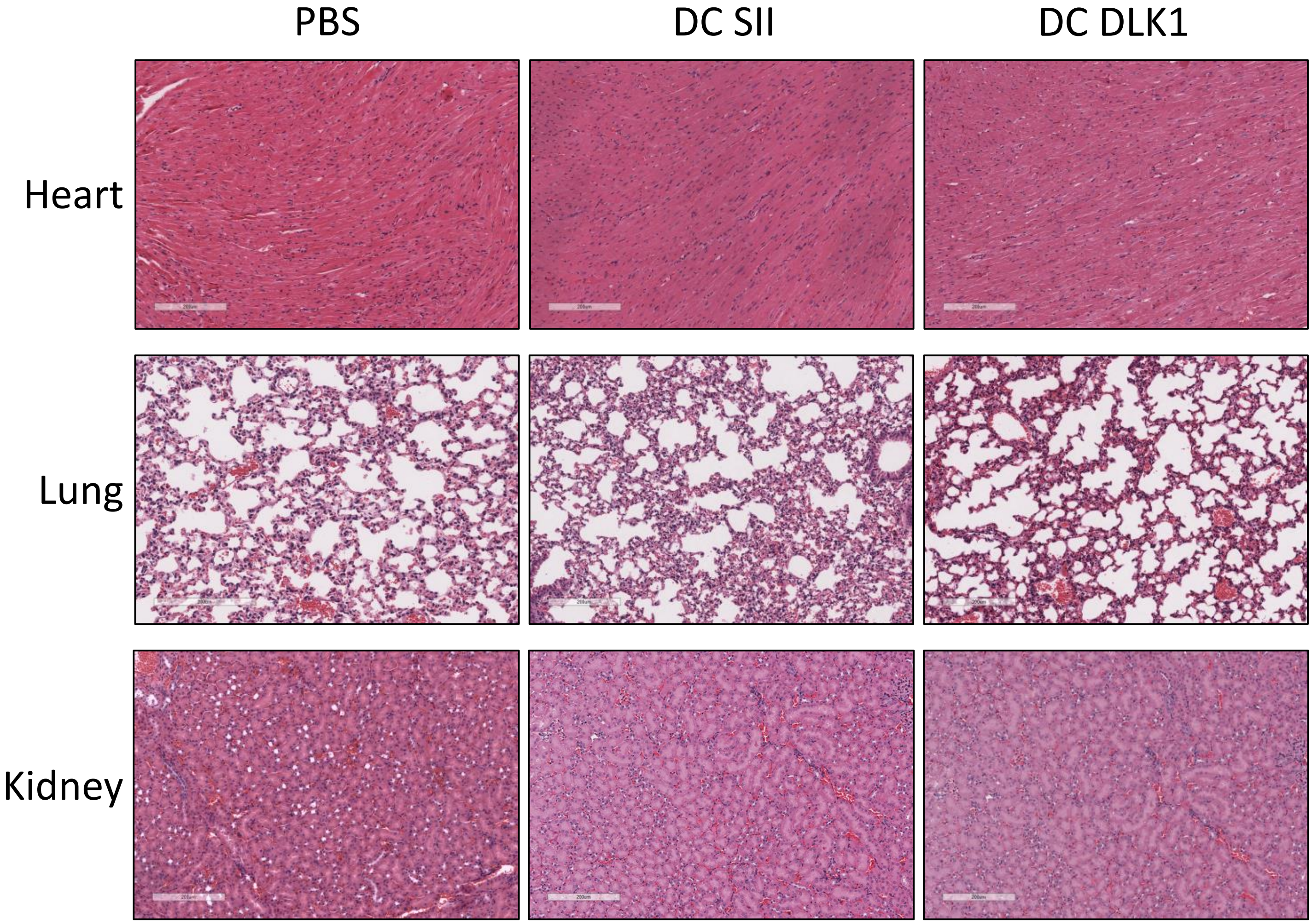

Supplementary Figure 6

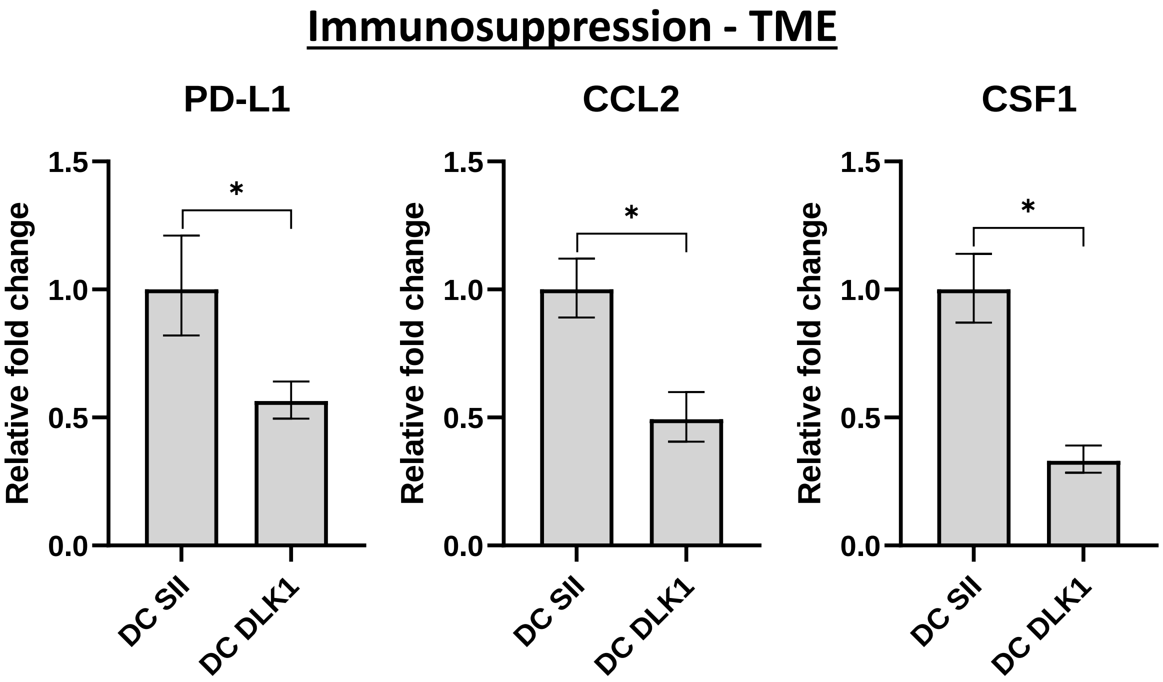

Supplementary Figure 7

(A)

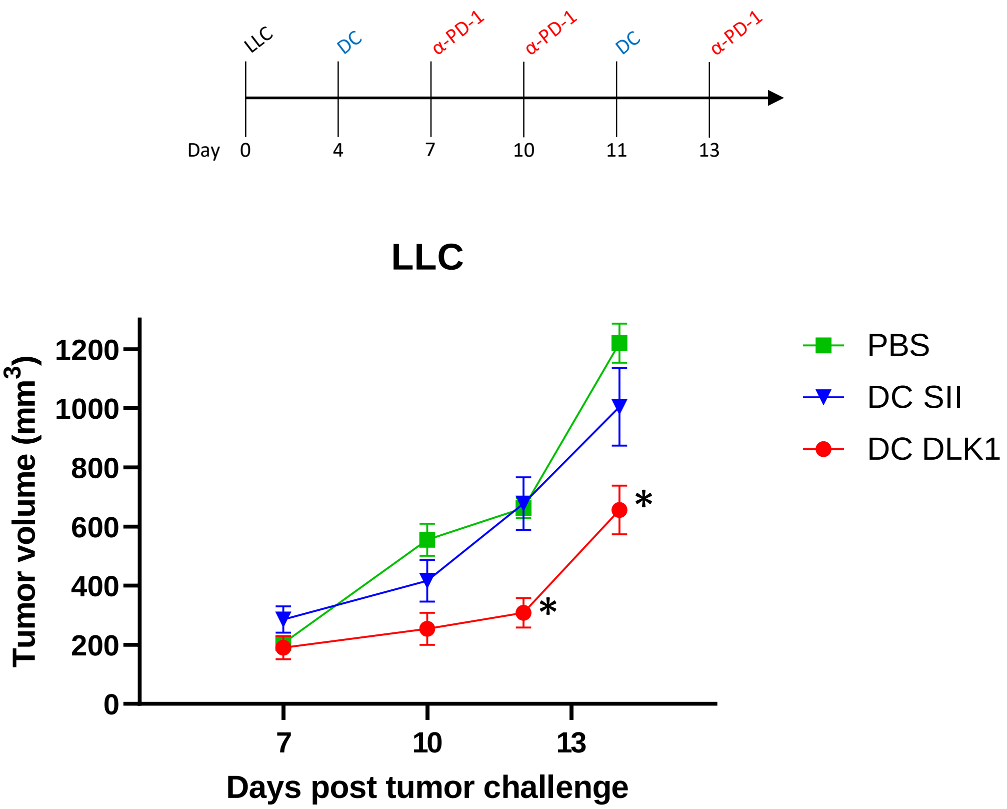

(B)

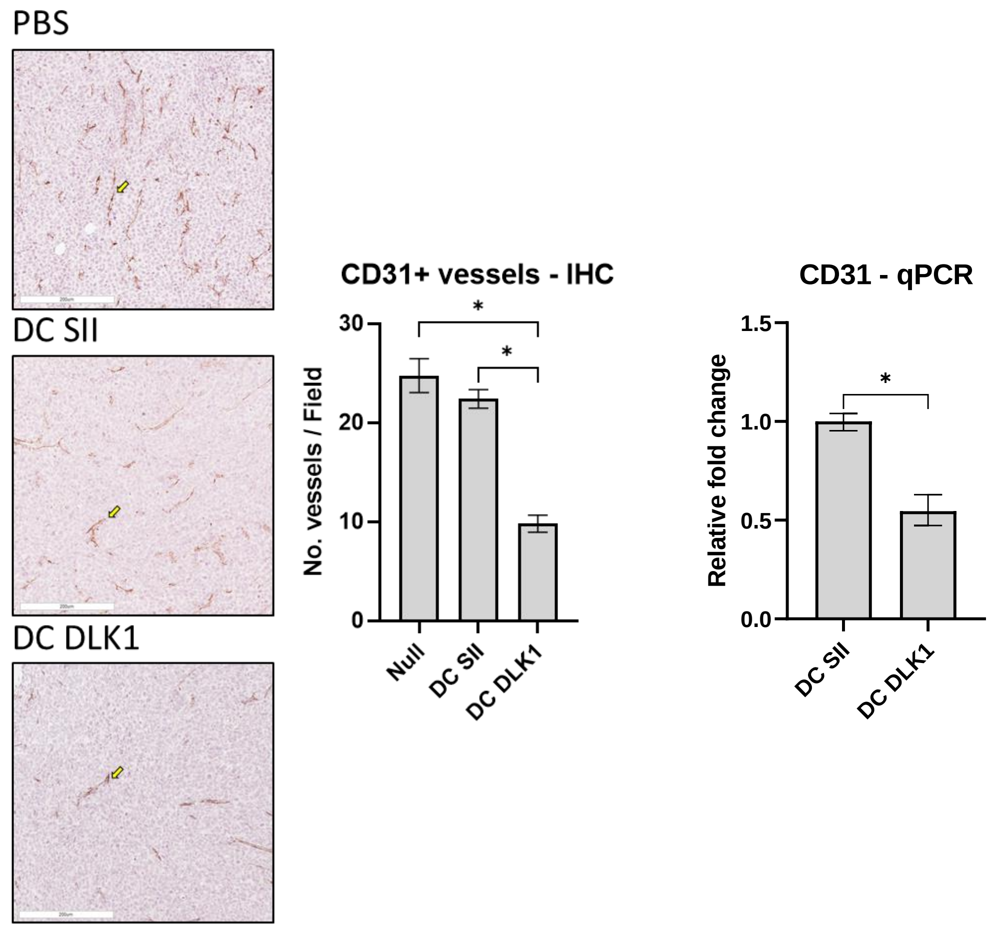

Supplementary Figure 8

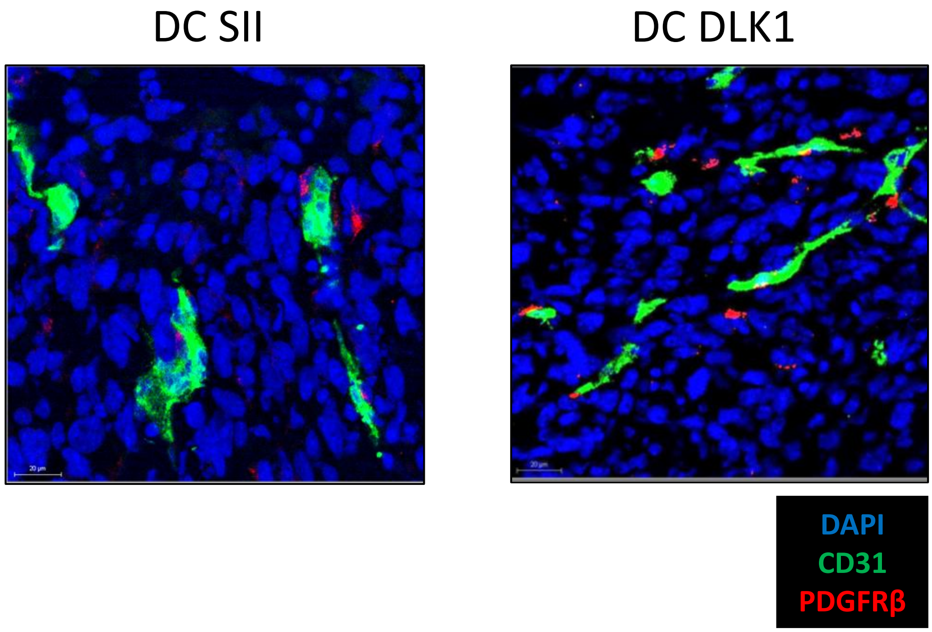

Supplementary Table 1

Supplementary Table 1: qPCR Primer Pairs

Target	Forward primer	Reverse primer
**GAPDH**	CATCACTGCCACCCAGAAGACTG	ATGCCAGTGAGCTTCCCGTTCAG
**CD31**	CCAAAGCCAGTAGCATCATGGTC	GGATGGTGAAGTTGGCTACAGG
**VEGFR2**	CGAGACCATTGAAGTGACTTGCC	TTCCTCACCCTGCGGATAGTCA
**PDGFR-beta**	GTGGTCCTTACCGTCATCTCTC	GTGGAGTCGTAAGGCAACTGCA
**DLK1**	TGGCTGTGTCAATGGAGTCTGC	CCACGCAAGTTCCATTGTTGGC
**IFN-gamma**	CAGCAACAGCAAGGCGAAAAAGG	TTTCCGCTTCCTGAGGCTGGAT
**PD-L1**	TGCGGACTACAAGCGAATCACG	CTCAGCTTCTGGATAACCCTCG
**CCL2**	GCTACAAGAGGATCACCAGCAG	GTCTGGACCCATTCCTTCTTGG
**CSF1**	GCCTCCTGTTCTACAAGTGGAAG	ACTGGCAGTTCCACCTGTCTGT
